# The Generation of CAR-Transfected Natural Killer T Cells for the Immunotherapy of Melanoma

**DOI:** 10.3390/ijms19082365

**Published:** 2018-08-11

**Authors:** Bianca Simon, Manuel Wiesinger, Johannes März, Kilian Wistuba-Hamprecht, Benjamin Weide, Beatrice Schuler-Thurner, Gerold Schuler, Jan Dörrie, Ugur Uslu

**Affiliations:** 1 Friedrich-Alexander-Universität Erlangen-Nürnberg (FAU), Department of Dermatology, Universitätsklinikum Erlangen, 91054 Erlangen, Germany; bianca.simon@uk-erlangen.de (B.S.); manuel.wiesinger@uk-erlangen.de (M.W.); johannes.maerz@uk-erlangen.de (J.M.); beatrice.schuler-thurner@uk-erlangen.de (B.S.-T.); gerold.schuler@uk-erlangen.de (G.S.); jan.doerrie@uk-erlangen.de (J.D.); 2 Friedrich-Alexander-Universität Erlangen-Nürnberg (FAU), Division of Genetics, Department of Biology, 91058 Erlangen, Germany; 3 Eberhard-Karls-Universität Tübingen, University Medical Center, Department of Dermatology, 72076 Tübingen, Germany; kilian.wistuba-hamprecht@uni-tuebingen.de (K.W.-H.); benjamin.weide@med.uni-tuebingen.de (B.W.)

**Keywords:** cancer, human, adoptive T-cell therapy, chimeric antigen receptor, T-cell receptor, immune escape, T cells, NKT cells

## Abstract

Natural killer T (NKT) cells represent a cell subpopulation that combines characteristics of natural killer (NK) cells and T cells. Through their endogenous T-cell receptors (TCRs), they reveal a pronounced intrinsic anti-tumor activity. Thus, a NKT cell transfected with a chimeric antigen receptor (CAR), which recognizes a tumor-specific surface antigen, could attack tumor cells antigen-specifically via the CAR and additionally through its endogenous TCR. NKT cells were isolated from peripheral blood mononuclear cells (PBMCs), expanded, and electroporated with mRNA encoding a chondroitin sulfate proteoglycan 4 (CSPG4)-specific CAR. The CAR expression on NKT cells and their in vitro functionality were analyzed. A transfection efficiency of more than 80% was achieved. Upon stimulation with melanoma cells, CAR-NKT cells produced cytokines antigen-specifically. Compared with conventional CAR-T cells, cytokine secretion of CAR-NKT cells was generally lower. Specific cytotoxicity, however, was similar with CAR-NKT cells showing a trend towards improved cytotoxicity. Additionally, CAR-NKT cells could kill target cells through their endogenous TCRs. In summary, it is feasible to generate CAR-NKT cells by using mRNA electroporation. Their CAR-mediated cytotoxicity is at least equal to that of conventional CAR-T cells, while their intrinsic cytotoxic activity is maintained. Thus, CAR-NKT cells may represent a valuable alternative to conventional CAR-T cells for cancer immunotherapy.

## 1. Introduction

Recently, chimeric antigen receptor (CAR)-T-cell therapy targeting the surface protein CD19 has shown impressive results in clinical trials. Complete responses were seen in about 80–90% of treated children and young adults with B-cell precursor acute lymphoblastic leukemia (ALL) that was refractory to standard therapy or in second or later relapse, leading to its approval in the United States [1]. Promising results and response rates were also observed in patients suffering from refractory large B-cell lymphoma after the failure of conventional therapy [2]. However, the same results were so far not observed in solid tumors [3,4]. In particular, the immunosuppressive tumor microenvironment in solid cancers is thought to negatively influence CAR-T-cell efficiency [3,4]. Another obstacle of CAR-T-cell therapy is the high rate of potential life-threatening side effects, including cytokine release syndrome (CRS), severe neurotoxicity, off-tumor on-target toxicities, and graft versus host disease (GvHD) caused by allogeneic cell therapy [5,6]. In order to improve CAR-T-cell therapy for solid tumors, as well as to deal with potential fatal side effects, new strategies need to be developed. This also brings the adoptive transfer of alternative cell populations (e.g., CAR-transfected natural killer T (NKT) cells) into focus [7].

NKT cells share characteristics of natural killer (NK) cells and T cells and represent an effective cell subpopulation in pathogen and tumor cell defense [8,9]. A crucial advantage of NKT cells over conventional T cells may be their pronounced intrinsic anti-tumor activity through their endogenous T-cell receptors (TCRs) [9,10], which are triggered by glycolipids presented via the monomorphic major histocompatibility complex (MHC) class Ib molecule CD1d on antigen-presenting cells [9,10]. Alpha-galactosylceramide (α-GalCer) represents a well-investigated CD1d ligand, which induces IL-4 and interferon-gamma (INFγ) production upon TCR ligation on NKT cells [11].

Cancer immunotherapy, with, for example, α-GalCer-loaded dendritic cells in order to activate NKT cells or adoptive transfer of NKT cells, has shown partial or complete responses, however, in mainly non-advanced stage cancer patients [12,13,14]. Contrary to conventional T-cell therapy, adoptive transfer of NKT cells is known to suppress GvHD development when clinically used in leukemia patients following bone marrow transplantation, while graft antitumor activity is still maintained [15,16]. Also, NKT cells are known to migrate into non-lymphoid tissues [17], indicating a beneficial effect especially for use in solid tumors. Thus, equipping NKT cells with CARs may represent a safer and equally effective approach for use in solid tumors compared with conventional CAR-T-cell therapy [18]. CAR-NKT cells, which recognize a tumor-specific surface antigen, could therefore attack tumor cells concomitantly in two different ways: on the one side, antigen-specifically via their CARs, and on the other side, via their intrinsic anti-tumor activity through their endogenous TCRs. Thus, CAR-NKT cells could still be active through their endogenous TCRs when tumor cells try to escape immune recognition, and their anti-tumor activity may be enhanced.

Options to transfer CARs into cells include a stable DNA-based receptor transfer and a transient RNA-based receptor transfer. Regarding potential fatal side effects, the RNA-based receptor transfer seems preferable over the standard DNA-based transduction [19,20,21]. Due to the absence of chromosomal integration and genetic alteration, RNA-based receptor expression, as well as receptor-mediated side effects, are transient [19,20,21]. Preclinical models revealed that T cells transfected with RNA (e.g., encoding mesothelin-, CD19-, or chondroitin sulfate proteoglycan 4 (CSPG4)-specific CARs) showed anti-tumor effects in vitro and in vivo [22,23,24]. The first patient reports of a phase I clinical trial using a mRNA-engineered anti-mesothelin CAR in progressive malignant pleural mesothelioma are already published [25].

The CSPG4 antigen (the melanoma-associated chondroitin sulfate proteoglycan, MCSP, or the high molecular weight melanoma-associated antigen, HMW-MAA) is expressed in 90% of melanoma lesions [26] and other malignancies (e.g., sarcomas and gliomas [27,28]). It plays a significant role in melanoma progression by influencing adhesion/spreading, migration, invasion, and metastasis [26], and thus, it may represent an effective target for use in the adoptive cell therapy of melanoma.

The aim of this study was the generation of CSPG4-specific CAR-NKT cells using RNA electroporation for potential use in cancer immunotherapy. The in vitro functionality of CAR-NKT cells (e.g., cytokine secretion efficiency, and cytolytic capacity) was analyzed after stimulation with melanoma cells expressing the target antigen and compared with that of conventional CAR-T cells. Additionally, the intrinsic cytolytic capacity of CAR-transfected NKT cells was analyzed after stimulation with α-GalCer-loaded CSPG4-negative target cells.

## 2. Results

### 2.1. NKT Cells Can Be Isolated from Peripheral Blood Mononuclear Cells (PBMCs) via Magnetic Activated Cell Sorting and Subsequently Expanded Using CD3 Monoclonal Antibody (OKT-3) and Interleukin-2 (IL-2)

Peripheral blood mononuclear cells (PBMCs) were derived from healthy donor blood, and NKT and CD8^+^ T cells were isolated via magnetic activated cell sorting (MACS). Both of the cell populations were then stimulated with OKT-3 and IL-2 for 10–11 days. After expansion, both of the cell populations were counted for comparison.

**Figure 1:**
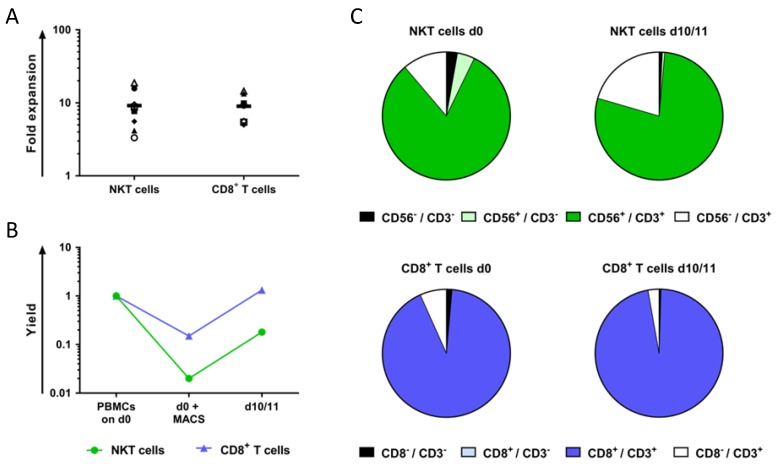
Natural killer T (NKT) cells can be isolated from peripheral blood mononuclear cells (PBMCs) and expanded by using CD3 monoclonal antibody (OKT-3) and interleukin-2 (IL-2). The NKT cells were derived from peripheral blood mononuclear cells (PBMCs) from healthy donors via magnetic-activated cell sorting (MACS). The CD8^+^ T cells were obtained from the NKT-negative cell fraction. Both of the cell populations were then stimulated with OKT-3 and IL-2 for 10–11 days. During expansion, IL-2 was added on days 2, 3, 5, and 7. The cells were counted on day 3 and re-adjusted to a concentration of 0.2 × 10^6^ cells per ml using fresh culture medium. On day 7, the total cell culture volume was doubled by adding fresh culture medium, and half of the total volume was transferred to a second culture flask. After expansion, the cells were counted, and the fold expansion was calculated by dividing final cell count by the initial number of cells at the beginning of stimulation. Prior to and after expansion, the NKT cells were double stained for CD56 and CD3, and the CD8^+^ T cells were double stained for CD8 and CD3. (**A**) The fold expansions of 7–8 independent experiments, each depicted as a different symbol, are shown. The resulted values are presented on a logarithmic scale. The mean values are shown as bars. (**B**) Yield of the NKT cells (green circles) and CD8^+^ T cells (blue triangles) before and after MACS isolation and final expansion with OKT-3 and IL-2 calculated per 1 × 10^6^ PBMCs. The resulted values are presented on a logarithmic scale. The average values of 6–8 independent experiments are displayed. The absolute values are listed in Table S1. (**C**) The composition of the cell subpopulations at the beginning and the end of expansion. The NKT cells were double stained for CD56 and CD3 (upper part), and the CD8^+^ T cells were double stained for CD8 and CD3 (lower part). The average values of 6–7 independent experiments are shown. The mean percentages of the different populations are listed in Table S2.

The fold expansion was calculated by dividing the final cell count by starting cell count after MACS. The NKT and CD8^+^ T cells both showed a 10-fold expansion (Figure 1A). In addition, the yield referred to 1 × 10^6^ PBMCs was determined directly after MACS-isolation and for day 10 or 11 (Figure 1B). The absolute values corresponding to Figure 1B are listed in Table S1. The isolation of the NKT and CD8^+^ T cells via MACS resulted in a yield of approximately 2% and 20% of total PBMCs, respectively (Figure 1B). Nevertheless, both of the cell populations expanded equally well, but due to the difference in the starting material, only the CD8^+^ T cells managed to regain the number of original PBMCs (Figure 1B).

On the first and the last day of expansion, the cells were stained for CD3 together with CD56 or CD8 to analyze different subpopulations. The MACS-isolated NKT cell fraction consisted, at the beginning of stimulation, of 81.57% CD56^+^CD3^+^ cells and maintained a purity of 78.05% of CD56^+^CD3^+^ cells after expansion (Figure 1C and Table S2). The CD8^+^ T cell fraction contained at the start of stimulation 91.88% CD8^+^CD3^+^ T cells, and this population increased to 96.72% following OKT-3-mediated expansion (Figure 1C and Table S2).

In summary, the fold expansion resulting from the OKT-3-mediated stimulation of the purified NKT cells was similar compared to the expansion of the CD8^+^ T cells, even if the percentage of the NKT cells in all PBMCs was lower compared with the CD8^+^ T cells. Furthermore, the activation of the NKT cells with OKT-3 and IL-2 led to an efficient expansion of the NKT^+^ cells with a predominant CD56^+^CD3^+^ population.

### 2.2. NKT Cells Can Be Efficiently Transfected with a CSPG4-Specific CAR Using mRNA Electroporation

To investigate whether mRNA electroporation is suitable to transfect NKT cells with a tumor-specific CAR, they were either electroporated without mRNA as controls (mock) or with mRNA coding for a CSPG4-specific CAR concomitant to CD8^+^ T cells. At different timepoints after electroporation, the receptor-transfected cells were stained with an antibody, specific for the Fc-spacer within the CARs, to analyze their expression levels.

**Figure 2:**
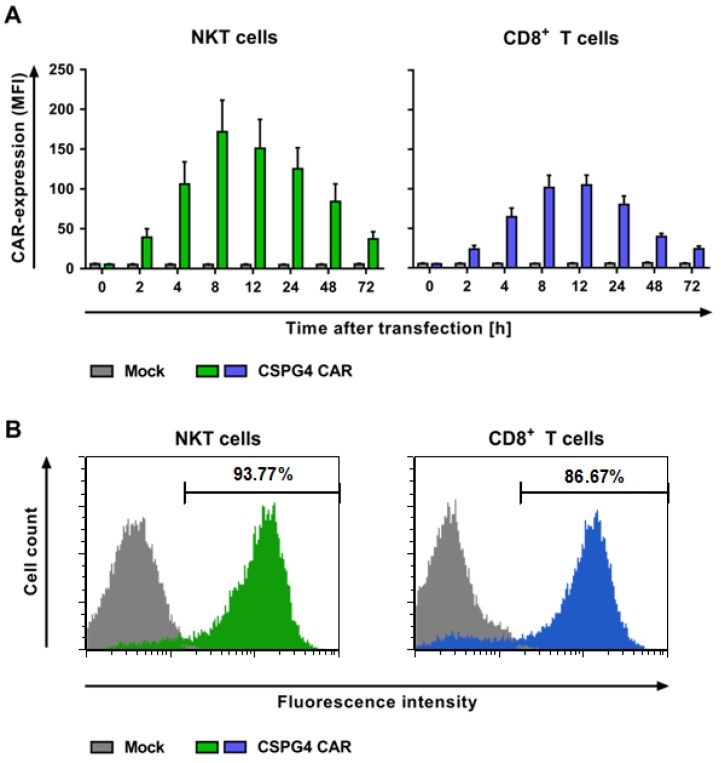
NKT cells can be efficiently transfected with a chondroitin sulfate proteoglycan 4 (CSPG4)-specific chimeric antigen receptor (CAR) using RNA electroporation. The NKT and CD8^+^ T cells were isolated and expanded as described in Figure 1. After 10–11 days, the cell populations were either transfected without mRNA (mock) as controls or with mRNA encoding a CSPG4-specific CAR. (**A**) The expression kinetics of the CAR-electroporated cells at indicated timepoints are shown. The CAR expression of the NKT (green bars) and CD8^+^ T cells (blue bars) was detected by using an anti-IgG1 antibody. The mock-transfected cells served as controls (grey bars). The data represent the average geometric mean values of 5–7 independent experiments with SEM. The *p*-values were calculated by unpaired Student’s *t*-test and are listed in Table S3. (**B**) Representative histograms at the peak of the CAR expression of both of the cell populations at 8 h after electroporation. The NKT cells are displayed as the green-filled histogram, and the CD8^+^ T cells as the blue-filled histogram. The mock-transfected cells served as controls (grey-filled histograms). The staining for the CAR expression was performed using the abovementioned antibody. The data of one representative out of 5–7 independent experiments are shown.

The CAR expression was detected on the surface of both the CAR-NKT and CD8^+^ CAR-T cells over the course of time. Figure 2A shows the mean fluorescence intensity (MFI) and Figure S1 the median CAR expression on the transfected NKT cells and CD8^+^ T cells. Mock transfected cells were used as controls and showed no CAR expression (Figure 2 and Figure S1).

The NKT cells showed the highest CAR expression 8 h after transfection, whereas the peak of the CAR expression was seen between 8 and 12 h after transfection for the CD8^+^ T cells (Figure 2 and Figure S1). The geometric mean, as well as the median values of the CAR staining, revealed a higher CAR expression for the NKT cells compared with the CD8^+^ T cells, without reaching statistical significance (Figure 2A and Figure S1 and Tables S3 and S4).

Further investigations of transfection efficacy displayed no significant differences in the percentages of dead cells between both of the cell populations and similar percentages of CAR-expressing cells (Figure S2 and Table S5).

Taken together, the NKT cells were successfully transfected with a melanoma-specific CAR via mRNA electroporation and revealed an equal transfection efficacy comparable to CD8^+^ T cells.

### 2.3. CAR-NKT Cells Specifically Produce Cytokines after Antigen Encounter

To compare the functionality of the CAR-NKT cells with the CD8^+^ CAR-T cells, the cytokine production of the receptor-transfected cells in response to a human melanoma cell line was analyzed. The cell populations were either electroporated with mRNA encoding a control CAR, specific for the carcinoembryonic antigen (CEA) or with mRNA coding for the CSPG4-specific CAR. The receptor-transfected cells were stimulated either with the TxB cell hybridoma T2.A1 (CSPG4^−^, CEA^−^) or the human melanoma cell line A375M (CSPG4^+^, CEA^−^), and the production of the pro-inflammatory cytokines interleukin-2 (IL-2), tumor necrosis factor (TNF), and interferon-gamma (IFNγ) was characterized.

**Figure 3:**
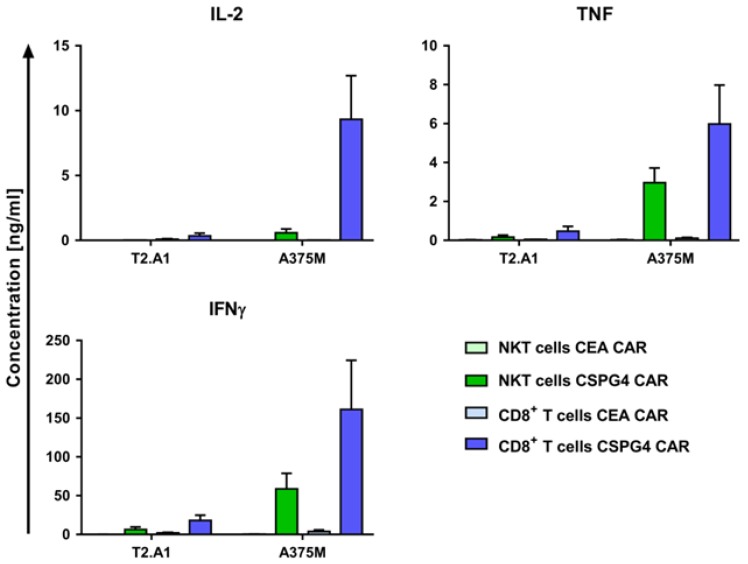
The CSPG4 CAR-transfected NKT cells secrete cytokines in an antigen-specific fashion. The NKT and CD8^+^ T cells were obtained as described above (see Figure 1). Following 10–11 days of expansion, the cells were either electroporated with mRNA coding for a carcinoembryonic antigen (CEA)-specific CAR or with mRNA encoding a CSPG4-specific CAR. The CEA CAR-transfected cells were used as controls. Then, 4 h after electroporation, the cells were co-cultured with target cells overnight. IL-2, tumor necrosis factor (TNF), and interferon-gamma (IFN-γ) production was measured in a cytometric bead array. As target cells, the TxB cell hybridoma T2.A1 (CSPG4^−^, CEA^−^) and the A375M melanoma cell line (CSPG4^+^, CEA^−^) were used. The receptor-transfected NKT cells are shown in light green bars (CEA CAR) and dark green bars (CSPG4 CAR), whereas electroporated the CD8^+^ T cells are displayed in light blue bars (CEA CAR) and dark blue bars (CSPG4 CAR). The average values of 4–7 independent experiments with SEM are shown. The *p*-values calculated by unpaired Student’s *t*-test are presented in Tables S6 and S7.

The CEA CAR-transfected cells revealed no unspecific cytokine production after co-cultivation with any of the target cells (Figure 3). Incubation with the negative target cell line T2.A1 showed only marginal induction of IL-2, TNF, and IFNγ for the CSPG4 CAR-transfected CD8^+^ T cells and even less for the CSPG4 CAR-transfected NKT cells (Figure 3).

An antigen-specific cytokine response was detected after co-cultivation of the cells transfected with the CSPG4-specific receptor with the melanoma cell line A375M, confirming the functionality of the CARs (Figure 3). The CD8^+^ CAR-T cells produced significant amounts of IL-2 and TNF and large quantities of IFNγ, almost reaching significance, compared with the negative targets and compared with the control CAR-transfected cells (Figure 3 and Tables S6 and S7). The CAR-NKT cells secreted smaller but significant levels of TNF and IFNγ and very little IL-2 (Figure 3 and Tables S6 and S7). We also addressed the production of the anti-inflammatory and the type 2 helper T cell (Th2) cytokines interleukin-4 (IL-4), interleukin-6 (IL-6), and interleukin-10 (IL-10) and detected no release of IL-10 and very low secretion of IL-4 and IL-6 (below 100 pg/mL; Figure S3).

Altogether, both of the CSPG4 CAR-transfected cell populations were able to produce significant amounts of pro-inflammatory cytokines antigen-specifically in response to human melanoma target cells, while the CD8^+^ CAR-T cells in general showed higher cytokine secretion compared with the CAR-NKT cells.

### 2.4. CAR-NKT Cells Lyse Human Melanoma Cells in an Antigen-Specific Manner

An essential feature of anti-cancer adoptive cell therapy is the ability of engineered cells to specifically lyse tumor cells. Thus, after receptor transfer, the cytolytic capacity of the NKT and CD8^+^ T cells was investigated for different effector-to-target ratios in a standard 4–6 h ^51^chromium release assay. Electroporation of a CEA-specific CAR was performed as a control to rule out unspecific activity of the CAR per se. The human lymphoma cell line T2.A1 (CSPG4^−^, CEA^−^) and the human melanoma cell line A375M (CSPG4^+^, CEA^−^) served as target cells.

**Figure 4:**
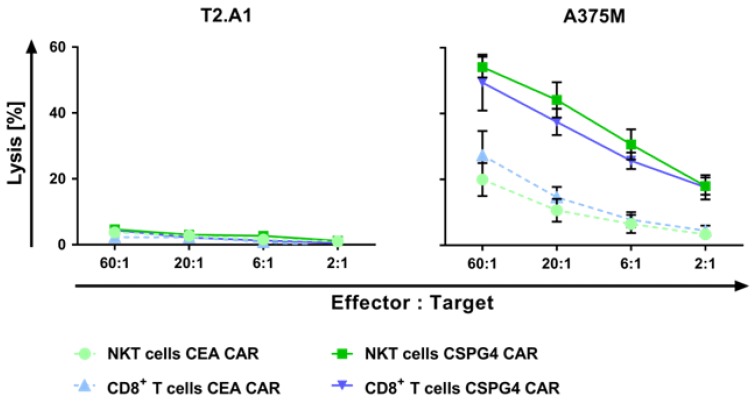
Melanoma cells are antigen-specifically lysed by the CSPG4 CAR-electroporated NKT cells. The NKT and CD8^+^ T cells were isolated and expanded as described for Figure 1. After 10–11 days, cell populations were either transfected with mRNA encoding a CEA-specific CAR or with mRNA coding for a CSPG4-specific CAR. The CEA CAR-transfected cells served as controls. Following overnight culture, the cytotoxicity of the receptor-transfected cells was determined in a standard 4–6 h ^51^chromium release assay. The TxB cell hybridoma T2.A1 (CSPG4^−^, CEA^−^) and the A375M melanoma cell line (CSPG4^+^, CEA^−^) were used as target cells. The percentage of lysed cells was calculated for indicated effector-to-target (E/T) ratios. The receptor-electroporated NKT cells are shown in light green dotted lines (CEA CAR) and dark green solid lines (CSPG4 CAR), whereas the transfected CD8^+^ T cells are displayed in light blue dotted lines (CEA CAR) and dark blue solid lines (CSPG4 CAR). The data represent the mean values of 4–7 independent experiments ± SEM. The *p*-values were calculated by unpaired Student’s *t*-test and are listed in Tables S8 and S9.

Little to no unspecific lysis of the negative target cell line T2.A1 was detected (Figure 4). The CEA CAR-transfected cells revealed a background cytotoxic activity of 20% for NKT cells and 25% for CD8^+^ T cells against the melanoma cells A375M (Figure 4).

The transfer of the CSPG4-specific CAR resulted in a strong lytic response for both T-cell populations, leading to approximately 50% lysed A375M target cells at the highest effector-to-target ratio (Figure 4). The CSPG4 CAR-transfected NKT cells showed a trend towards higher cytotoxicity compared with the CD8^+^ CAR-T cells, without reaching statistical significance (Figure 4 and Table S8). This could be due to the intrinsic cytolytic capacity of the NKT cells. The comparison of the mock-transfected NKT and CD8^+^ T cells revealed a trend towards a higher intrinsic lysis of A375M by the NKT cells, while very little endogenous cytotoxicity against T2.A1 was observed (Figure S4).

The statistical investigation concerning the antigen-specificity of lysis revealed a highly significant increase in the cytolytic capacity of both of the cell populations after co-cultivation with the A375M cells compared with stimulation with the T2.A1 cells for all effector-to-target ratios (Table S9).

In summary, the receptor-transfected NKT cells are capable of specifically lysing human melanoma cells after being equipped with CSPG4-specific CARs. Moreover, these cells eliminated target cells to an at least equal extent as the conventional CD8^+^ CAR-T cells.

### 2.5. CAR-NKT Cells Retain Their Intrinsic Lytic Capacity Against α-GalCer-Loaded Jurkat Cells Expressing CD1d

The endogenous TCR of a NKT cell functions through the recognition of lipid antigens presented via a conserved and non-polymorphic MHC class Ib molecule, called CD1d [10,11]. The glycolipid α-GalCer is known to be a potent activator of NKT cells and leads to a strong immune response [10,11]. The human T-cell leukemia cell line Jurkat constitutively expresses the CD1d molecule, and when pulsed with α-GalCer, it operates as an ideal target to examine the intrinsic cytotoxicity of NKT cells [29].

Thus, the endogenous cytolytic capacity of the CAR-NKT cells was analyzed and compared with the CD8^+^ CAR-T cells after stimulation with the target cell line Jurkat (CD1d^+^, CSPG4^−^, CEA^−^) either unpulsed or α-GalCer-loaded in a standard 4–6 h ^51^chromium release assay. The mock-transfected T cells served as negative controls.

**Figure 5:**
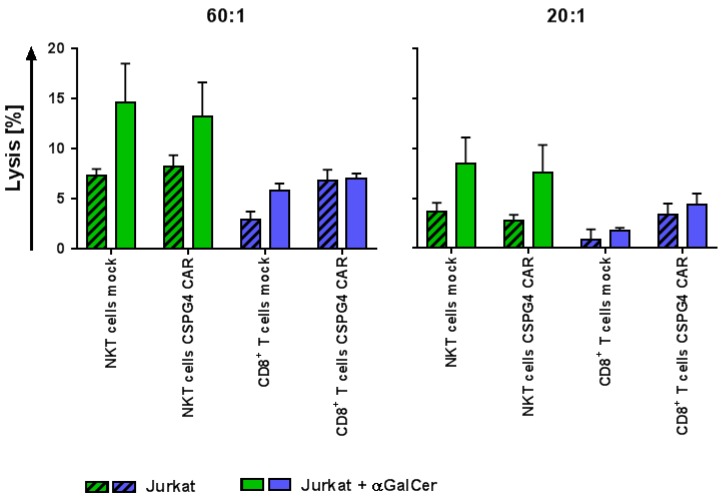
The CSPG4 CAR-transfected NKT cells maintain their intrinsic cytotoxicity against α-GalCer-loaded Jurkat cells expressing CD1d. The NKT and CD8^+^ T cells were obtained as described above (see Figure 1). Following 10–11 days of expansion, the cells were either electroporated without mRNA (mock) or with mRNA encoding a CSPG4-specific CAR. The mock-transfected cells were used as controls. After overnight culture, the cytolytic capacity of receptor-transfected cells was examined in a standard 4–6 h ^51^chromium release assay after stimulation with Jurkat T-cell leukemia cells (CD1d^+^, CSPG4^−^, CEA^−^), either unpulsed or α-GalCer-loaded. The percentage of lysed cells was calculated for indicated effector-to-target (E/T) ratios. The lysis of unpulsed Jurkat cells is shown in hatched bars, and the cytotoxicity of α-GalCer-loaded Jurkat cells is displayed in filled bars. The NKT cells are shown in green bars (mock and CSPG4 CAR), whereas the CD8^+^ T cells are displayed in blue bars (mock and CSPG4 CAR). The average values of 4 independent experiments with SEM are shown. The *p*-values calculated by unpaired Student’s *t*-test are presented in Tables S10 and S11.

The mock-electroporated CD8^+^ T cells revealed little background lysis, whereas a significantly higher cytotoxicity was seen with the mock-transfected NKT cells at the highest effector-to-target ratio when stimulated with unpulsed Jurkat cells (Figure 5 and Figure S5 and Table S10). The killing by the CSPG4 CAR-transfected NKT cells occurred to a similar extent compared to the receptor-transfected CD8^+^ T cells when unpulsed Jurkat cells were used (Figure 5 and Figure S5 and Table S10). The α-GalCer-loading of Jurkat cells displayed an increased cytolytic capacity of both the NKT cell conditions (mock and CSPG4 CAR), while no changes for the electroporated CD8^+^ T cells was observed, except for the mock-transfected T cells at the highest effector-to-target ratio (Figure 5 and Figure S5 and Tables S10 and S11). The killing capacity of the mock-electroporated NKT cells did not differ from that of the CSPG4 CAR-transfected NKT cells (Figure 5 and Figure S5 and Table S10).

Taken together, the receptor-transfected NKT cells appeared to maintain their intrinsic lytic activity against the human T cell leukemia cell line Jurkat and even increased their killing after the α-GalCer-loading of the target cells, whereas the electroporated CD8^+^ T cells only showed unspecific background lysis.

## 3. Discussion

The use of CAR-T cells is one of the big new success stories of cancer immunotherapy. Nevertheless, especially in solid tumors, different issues remain to be addressed, with loss of antigen being one of them [30]. Because CAR therapy exerts selective pressure on the tumor, the logical consequence is an outgrowth of tumor cells that are negative for the target antigen. Alternative effector cells of the immune system transfected with CARs may counteract this process, as they could, for instance, attack antigen-negative tumor cells via other intrinsic receptors, thus negating their selective advantage [7].

The number and functionality of NKT cells in the blood or the tumor was shown to correlate with better survival in a variety of human cancers [9]. In addition, the in vivo activation of these cells led to promising clinical results [31]. In a phase 1 trial with melanoma patients, the use of NKT cells was shown to be feasible [14]. However, this trial further demonstrated that this treatment regimen needs to be improved to yield clinical efficiency [14]. Thus, methods to improve the tumor-specific activation of these cells were developed, and the combination of this cell population’s intrinsic anti-tumor activity with CAR-mediated specificity was the next logical step [18,32]. NKT cells retrovirally transduced with CARs against GD2 and CD19 already proved to be effective in preclinical experiments, indicating both CAR-mediated and endogenous anti-tumor activity [18,32]. To our knowledge, mRNA-based CAR transfer was so far not tested for NKT cells. In this study, we could observe that NKT CAR-mediated cytotoxicity was similar to CAR-transfected conventional T cells after electroporation, while their endogenous reactivity towards αGalCer-loaded targets was maintained. Not all NKT cells are CD1d-restricted [33], which might explain the lower intrinsic cytotoxicity compared with the previously published studies [34]. Nevertheless, we were able to generate a new and potentially powerful tool against cancer, combining features of both worlds, the adaptive and the innate immunity.

In this manuscript, we provide a first approach for the use of NKT cells for transient RNA-based CAR transfer in a preclinical in vitro setting. The next step towards the potential use of these CAR-NKT cells in a clinical setting will be the use of the engineered NKT cells in cancer mouse models. In addition, further analysis regarding relevant target molecules, which are ideally only expressed by cancer cells rather than by non-malignant cells, is favorable.

Another major reason for the use of alternative strategies to conventional CAR-T cells is the high risk of potential side effects (e.g., a massive release of pro-inflammatory cytokines, the so-called cytokine release syndrome (CRS)) [5]. The key cytokine in this process appears to be IL-6, and thus, the blocking of the IL-6 receptor with the monoclonal antibody tocilizumab is the approved regimen to treat CRS [35]. Our CAR-transfected NKT cells produced small quantities of IL-6 and clearly less than the CAR-transfected CD8^+^ T cells, indicating that the risk of CRS is reduced. Other cytokines involved in CRS (e.g., TNF and IFNγ) were also produced in smaller amounts by the CAR-NKT cells compared with the CD8^+^ CAR-T cells, but still in sufficient quantities. This is favorable, because these cytokines contribute to the anti-tumor activity of the engineered cells. The most important function in cancer therapy, however, is the direct lytic activity of the transfected cells against the tumor cells, which was similar for the CAR-NKT cells and the CAR-transfected CD8^+^ T cells in this study. In summary, this indicates that our CAR-NKT cells may be a safer but similarly efficient alternative to conventional CAR-T cells.

Another drawback of CAR-T cells are off-tissue effects, depending on the used target antigen [6]. In CD19-CAR-T cell therapy, which showed impressive clinical results in hematological malignancies, the loss of healthy CD19^+^ cells can be compensated by supplementing i.v. immunoglobulins. For other antigens, this can be more dangerous, as shown in a clinical trial with a CAR specific for carbonic anhydrase IX, which caused severe antigen-specific liver toxicity [36]. However, the development of these symptoms was of comparably slow onset, taking weeks to months until manifestation. By using transient transfection, this risk could be reduced, as treatment can be stopped at any time, and CAR expression will cease within 72 h.

In our study, the MACS-isolated NKT cell fraction consisted, at the beginning of stimulation, of approximately 81.57% CD56^+^CD3^+^ cells and maintained a purity of approximately 78.05% CD56^+^CD3^+^ cells after expansion. After MACS isolation, we also observed a CD3^+^CD56^−^ population, indicating that a subpopulation of NKT cells does not express CD56, which is already known and described in the literature [33]. Thus, the total amount of pure NKT cells is most likely higher in our study. Further analysis and investigations should, however, be performed toward the potential use of CAR-transfected NKT cells in a clinical setting to increase the total amount of NKT cells after activation and expansion.

Transferring our findings from bench to bedside could lead to a treatment strategy summarized as follows: (i) generation of a large amount of NKT cells (feasibility was shown in several studies [37,38,39], even though further modifications to improve NKT purity should be performed); (ii) electroporation with a CAR-mRNA encoding for a suitable antigen (e.g., CSPG4 for melanoma); (iii) cryopreservation (we could not find impairments on the viability of NKT cells through the latter); and (iv) reinfusion into the patient in repeated injections.

Taken together, it is feasible to transfect NKT cells with a tumor-antigen specific CAR by using mRNA electroporation. While CAR-mediated cytokine secretion of NKT cells was lower, specific cytotoxicity was at least equal to that of conventional CAR-T cells. Additionally, their intrinsic cytotoxic activity was maintained after mRNA-based receptor transfer, suggesting that these cells might contribute to cancer immunosurveillance even after losing their transduced CARs. Thus, CAR-NKT cells may represent a valuable alternative to conventional CAR-T cells for future developments of novel treatment strategies, as alternatives or supplementation to the existing treatment regimen of melanoma.

## 4. Materials and Methods

### 4.1. Cells and Reagents

Healthy donor blood was obtained after informed consent and approval by the institutional review board (Ethikkommission of the Friedrich-Alexander-Universität Erlangen-Nürnberg; reference number: 65_16 B; 16-03-2016). For the isolation of PBMCs, density centrifugation was performed using a Lymphoprep reagent (Axis-Shield, Oslo, Norway). Magnetic activated cell sorting (MACS) was used to successively collect NKT and CD8^+^ T cells according to the manufacturer’s instruction (human CD3^+^ CD56^+^ NKT Cell Isolation Kit and human CD8 MicroBeads; Miltenyi, Bergisch-Gladbach, Germany). The purified cells were adjusted to a concentration of 1 × 10^6^ cells/mL for expansion (see below) in R10 medium consisting of RPMI 1640 (Lonza, Basel, Switzerland) supplemented with 2 mM l-glutamine (Lonza), 100 IU/mL penicillin (Lonza), 100 mg/mL streptomycin (Lonza), 10% (*v*/*v*) heat-inactivated fetal calf serum (PAA, GE healthcare, Piscataway, NY, USA), 2 mM HEPES (PAA, GE Healthcare, Little Chalfont, UK), and 2 mM β-mercaptoethanol (Gibco, Life Technologies, Carlsbad, CA, USA).

As target cells, the TxB cell hybridoma T2.A1 (CSPG4^−^ and CEA^−^ [40,41]; a kind gift from Erwin Schulz, Nuremberg), the melanoma cell line A375M (CSPG4^+^ and CEA^−^ [40,42]; a kind gift from Corlien Aarnoudse, Leiden, The Netherlands; ATCC CRL-3223), and the T-cell leukemia cell line Jurkat (CSPG4^−^, CEA^−^ and CD1d^+^ [29]; a kind gift from Georg Fey, Erlangen) were used. Additionally, Jurkat cells were pulsed with 200 ng/mL alpha-galactosylceramide (α-GalCer) overnight for the indicated experiments. The target cells were cultured in R10 medium prior to co-incubation with the effector cells.

### 4.2. Expansion of T Cells

MACS-purified NKT cells and CD8^+^ T cells were expanded according to an in-house established expansion protocol [43]. In brief, at the beginning of expansion, the cells were adjusted to 1 × 10^6^ cells/mL with R10 medium and stimulated with 0.1 µg/mL anti-CD3 antibody OKT-3 (Orthoclone OKT-3; Janssen-Cilag, Neuss, Germany). Interleukin-2 (1000 IU/mL; Proleukin; Novartis, Nuremberg, Germany) was added on day 0, 2, 3, 5, and 7. On day 3, the cells were counted and further cultured at 0.2 × 10^6^/mL with fresh R10 medium. The cells were thoroughly resuspended and doubled in culture volume by adding new R10 medium on day 7. After 10 or 11 days, the cells were harvested for further experiments.

### 4.3. Flow Cytometric Analyses of Phenotypic Parameters

In order to analyze the cellular composition of the cell populations before and after expansion, the cells were stained using an anti-CD3 antibody (Clone: UCHT1; BD Biosciences, Franklin Lakes, NJ, USA) in combination with either an anti-CD56 antibody (Clone: NCAM16.2; BD Biosciences) or an anti-CD8 antibody (Clone: SK1; BD Biosciences). Isotype-stained cells served as controls. Additionally, all the cells were stained with an anti-7-AAD antibody (BD Biosciences) to exclude nonviable cells. Prior to staining, the cells were cryopreserved as previously described [19]. Immunofluorescence was measured via a FACS Calibur (BD Biosciences, Heidelberg, Germany) equipped with CellQuest Pro software (BD Biosciences). The data were analyzed using FCS Express, version 5 (DeNovo Software, Glendale, CA, USA).

### 4.4. RNA Production and Transfection

The mRNA used for electroporation was generated and purified using the mMESSAGE mMACHINE T7 Ultra Transcription Kit (Life Technologies, Carlsbad, CA, USA) and the RNeasy Kit (Qiagen) according to the manufacturers’ instructions. T cells were either transfected with RNA coding for the CSPG4-specific CAR (MCSP_HL_ CD28-CD3ζ) [22] or with RNA encoding the carcinoembryonic antigen (CEA)-specific CAR (CEA CD28-CD3ζ) [44] using a GenePulser Xcell system (Bio-Rad, Hercules, CA, USA) with the square-wave protocol at 500 V for 5 ms. After transfection, T cells were immediately transferred to R10 medium.

### 4.5. Surface Expression of Transfected Receptors

Receptor expression on the cell surface of transfected T cells was analyzed via flow cytometry at indicated timepoints after electroporation. The cells were cryopreserved prior to staining. The CAR was stained with a goat-F(ab’)2 anti-human IgG antibody (Southern Biotech, Birmingham, AL, USA) directed against the extracellular IgG1 CH_2_CH_3_ CAR-domain. The detailed procedure of the cell surface staining was previously described [45]. Immunofluorescence was measured via a FACScan cytofluorometer (BD Biosciences) equipped with CellQuest software (BD Bioscience). The data were analyzed using FCS Express software, version 5 (DeNovo Software, Glendale, CA, USA).

### 4.6. Cytokine Production

Cytokine secretion of T cells was assayed as previously described [46]. In short, transfected T cells were stimulated overnight with ultraviolet (UV)-irradiated (0.005 J/cm^2^) target cell lines T2.A1 and A375M at a 1:1 ratio. The cytokine concentrations in the supernatants were determined using the Th1/Th2 Cytometric Bead Array Kit II (BD Bioscience) according to the manufacturer’s instructions. Immunofluorescence was measured via the FACSCanto II (BD Biosciences) equipped with FACSDiva software (BD Bioscience). The data were analyzed using FCS Express software, version 5 (DeNovo Software).

### 4.7. Cytotoxicity

The cytolytic capacity of the transfected T cells was examined with a standard 4–6 h ^51^chromium-release assay 24 h after electroporation as described before [46]. In brief, target cell lines T2.A1, A375M, and Jurkat (either unloaded or α-GalCer-pulsed) were labelled with 20 µCi of Na_2_^51^CrO_4_/10^6^ cells (Perkin Elmer, Waltham, MA, USA) for 1 h. The transfected T cells were added in different effector cells to target cell ratios (E/T, respectively): 60:1, 20:1, 6:1, and 2:1. The chromium release in the supernatants was measured using the Wallac 1450 MicroBeta plus Scintillation Counter (Wallac, Turku, Finland). The percentage of lysis was calculated using the following formula: [(measured release − background release)]/[(maximum release − background release)] × 100%.

### 4.8. Figure Preparation and Statistical Analysis

The graphs were created and statistical analysis was performed using GraphPad Prism, version 7 (GraphPad Software, La Jolla, CA, USA). The *p*-values were analyzed using the unpaired Student’s *t*-test, assuming a Gaussian distribution. * indicates *p* ≤ 0.05, ** indicates *p* ≤ 0.01, *** indicates *p* ≤ 0.001, and **** indicates *p* ≤ 0.0001.

## Supplementary Materials

Supplementary materials can be found at http://www.mdpi.com/1422-0067/19/8/2365/s1.

## Abbreviations

αGalCer	alpha-galactosylceramide
ALL	acute lymphoblastic leukemia
CAR	chimeric antigen receptor
CEA	carcinoembyronic antigen
CRS	cytokine release syndrome
CSPG4	chondroitin sulfate proteoglycan 4
GvHD	graft versus host disease
HMW-MAA	high molecular weight-melanoma associated antigen
IFNγ	interferon-gamma
IL	Interleukin
MACS	magnetic-activated cell sorting
MCSP	melanoma-associated chondroitin sulfate proteoglycan
NK cells	natural killer cells
NKT cells	natural killer T cells
PBMCs	peripheral blood mononuclear cells
TCR	T-cell receptor
TNF	tumor necrosis factor
