# Supplementary material for: The Generation of CAR-Transfected Natural Killer T Cells for the Immunotherapy of Melanoma
Source: Int J Mol Sci. 2018 Aug 11;19(8):2365. doi: 10.3390/ijms19082365 (PMC6121949; doi:10.3390/ijms19082365)
Supplement: Supplementary file 1 [file ijms-19-02365-s001.pdf]

International Journal of
Molecular Sciences

Supplemental Materials

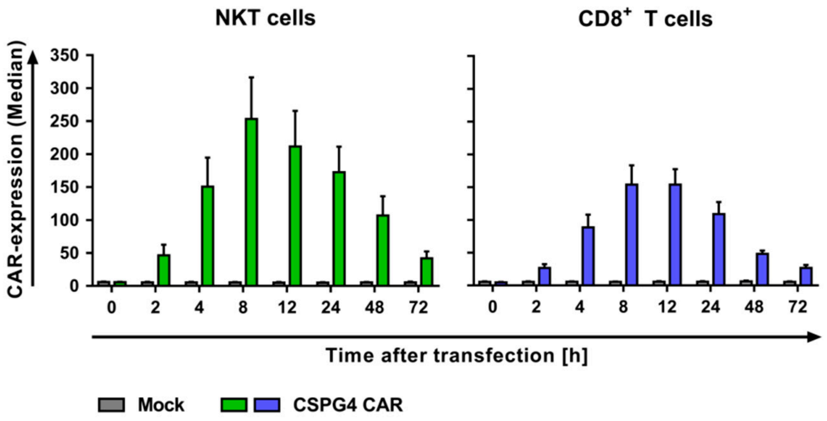

Figure S1. NKT cells can be efficiently transfected with a CSPG4-specific CAR using RNA-electroporation. The NKT and CD8+ T cells were isolated and expanded as described for Figure 1. After 10–11 days, the cell populations were either transfected without mRNA (mock) as controls or with mRNA encoding a CSPG4-specific CAR. The expression kinetics of the CAR-electroporated cells at indicated timepoints are shown. The CAR expression of the NKT (green bars) and CD8+ T cells (blue bars) was detected by using an anti-IgG1 antibody. The mock-transfected cells served as controls (grey bars). The data represent the average median values of 5–7 independent experiments with SEM. The p-values were calculated by unpaired Student's *t*-test and are listed in Table S4.

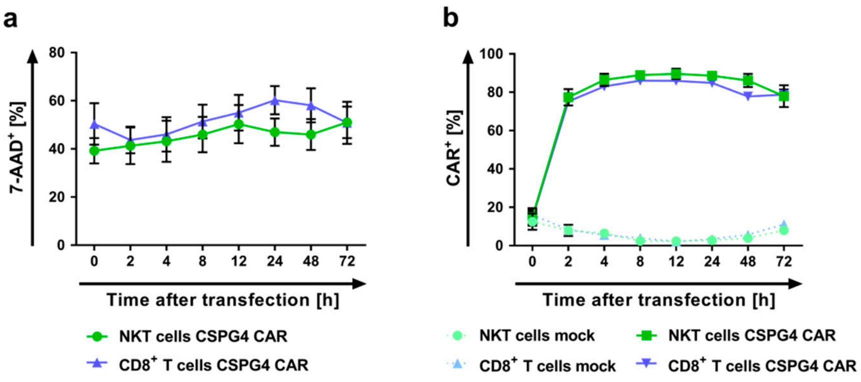

Figure S2. The CAR-electroporated NKT cells show similar percentages of dead cells and CAR-positive cells compared to the CAR-transfected CD8+ T cells. The NKT and CD8+ T cells were obtained as described in Figure 1. Following 10–11 days of expansion, the cells were either electroporated without mRNA as controls (mock) or with mRNA encoding a CSPG4-specific CAR. (**a**) The expression kinetics of 7-AAD-positive cells at indicated timepoints after transfection. The receptor-transfected NKT cells are shown in green lines, whereas the electroporated CD8+ T cells are displayed in blue lines. The data represent the average percentages of 5–7 independent experiments ± SEM. (**b**) The CAR-positive cells at indicated timepoints after transfection. The NKT cells are shown in light green dotted lines (mock) and dark green solid lines (CSPG4 CAR), whereas the CD8+ T cells are displayed in light blue dotted lines (mock) and dark blue solid lines (CSPG4 CAR). The average percentages of 5–7 independent experiments ± SEM are shown. The p-values were calculated by unpaired Student's t-test and are listed in Table S5.

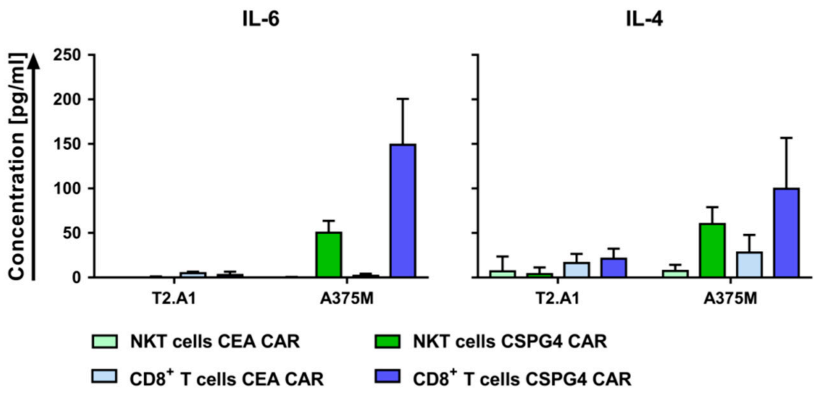

Figure S3. The CSPG4 CAR-transfected NKT cells secrete lower amounts of IL-6 and IL-4 compared with the CD8+ T cells. The NKT and CD8+ T cells were obtained as described in Figure 1. Following 10–11 days of expansion, the cells were either electroporated with mRNA coding for a CEA-specific CAR or with mRNA encoding a CSPG4-specific CAR. The CEA CAR-transfected cells were used as controls. Then, 4 h after electroporation, the cells were co-cultured with target cells overnight. IL-6 and IL-4 production was measured in a cytometric bead array. As target cells, the TxB cell hybridoma T2.A1 (CSPG4-, CEA-) and the A375M melanoma cell line (CSPG4+, CEA-) were used. The receptor-transfected NKT cells are shown in light green bars (CEA CAR) and dark green bars (CSPG4 CAR), whereas the electroporated CD8+ T cells are displayed in light blue bars (CEA CAR) and dark blue bars (CSPG4 CAR). The average values of 4–7 independent experiments with SEM are shown.

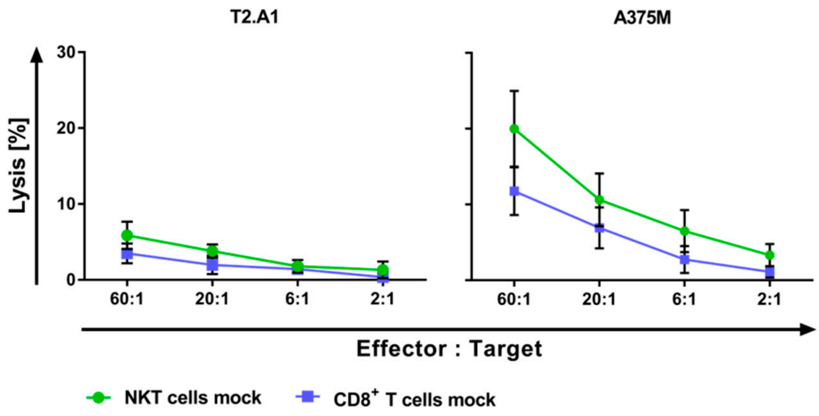

Figure S4. The NKT cells show a trend towards higher intrinsic lytic capacity towards A375M target cells compared with the CD8+ T cells. The NKT and CD8+ T cells were isolated, expanded, and transfected as described in Figure 1. Following overnight culture, cthe ytotoxicity of cells was determined in a standard 4–6 h ^{51}chromium release assay. The TxB cell hybridoma T2.A1 (CSPG4-, CEA-) and the A375M melanoma cell line (CSPG4+, CEA-) were used as target cells. The percentage of lysed cells was calculated for the indicated effector-to-target (E/T) ratios. The NKT cells are shown in dark green solid lines, whereas the CD8+ T cells are displayed in dark blue solid lines. The data represent the mean values of 4–7 independent experiments ± SEM.

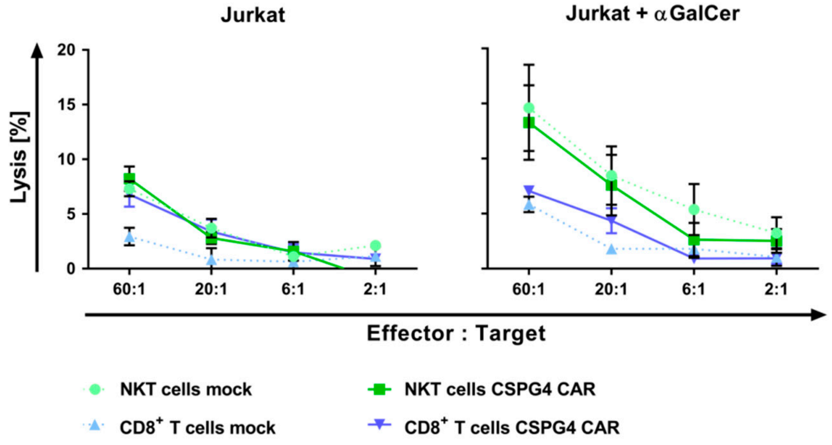

Figure S5. The NKT cells maintain their intrinsic cytolytic capacity against α-GalCer-loaded Jurkat cells expressing CD1d after receptor transfection. The NKT and CD8+ T cells were isolated and expanded as described in Figure 1. After 10–11 days, the cell populations were either electroporated without mRNA as controls (mock) or with mRNA encoding a CSPG4-specific CAR. Following overnight culture, the cytotoxicity of the receptor-transfected T cells was determined in a standard 4–6 h ^{51}chromium release assay after stimulation with the target cells. As the target cells, the Jurkat T-cell leukemia cell line (CD1d+, CSPG4-, CEA-) was used either unloaded or α-GalCer loaded. The percentage of lysed cells was calculated for the indicated effector-to-target (E/T) ratios. The NKT cells are displayed in light green dotted lines (mock) and dark green solid lines (CSPG4 CAR), whereas the CD8+ T cells are shown in light blue dotted lines (mock) and dark blue solid lines (CSPG4 CAR). The data represent the mean values of 4 independent experiments ± SEM. The p-values were calculated by unpaired Student's t-test and are listed in Tables S10 and S11.

Table S1. Absolute values[1] corresponding to Figure 1B.

Conditions NKT cells (*n* = 8)	PBMCs on day 0	day 0 + MACS	day 10/11
donor 1	203.7	6.0	78.8
donor 2	400.0	7.6	94.0
donor 3	236.0	3.7	12.5
donor 4	268.0	3.3	10.0
donor 5	445.0	5.5	25.0
donor 6	320.0	7.2	47.0
donor 7	460.0	11.2	76.8
donor 8	540.0	2.3	17.5
Conditions $CD8^+$ T cells (*n* = 6)	PBMCs on day 0	day 0 + MACS	day 10/11
donor 1	121.0	26.7	67.0
donor 2	165.0	32.3	46.6
donor 3	100.0	9.2	40.0
donor 4	155.0	29.0	50.0
donor 5	200.0	22.0	98.8
donor 6	243.0	24.0	54.5

[1] Absolute numbers are shown as 1×10^6 cells.

Table S2. Comparative fluorescence-activated cell sorting (FACS) analysis of NKT and $CD8^+$ T cell subpopulations before and after expansion [1,2].

Conditions NKT cells (*n* = 7)	day 0	day 10/11
$CD56^-$ / $CD3^-$	2.80	0.83
$CD56^+$ / $CD3^-$	4.40	0.61
$CD56^+$ / $CD3^+$	81.57	78.05
$CD56^-$ / $CD3^+$	11.24	20.51
Conditions $CD8^+$ T cells (n = 6)	day 0	day 10/11
$CD8^-$ / $CD3^-$	1.30	0.33
$CD8^+$ / $CD3^-$	0.12	0.16
$CD8^+$ / $CD3^+$	91.88	96.72
$CD8^-$ / $CD3^+$	6.70	2.79

[1] Data corresponding to Figure 1C; [2] Average percentages are shown.

Table S3. *p*-values [1] corresponding to Figure 2A.

Conditions	Timepoints							
	**0 h**	**2 h**	**4 h**	**8 h**	**12 h**	**24 h**	**48 h**	**72 h**
NKT cells mock vs NKT cells CSPG4 CAR	ns	**	**	**	**	***	**	**
NKT cells mock vs $CD8^+$ T cells mock	ns	ns	ns	ns	ns	ns	ns	ns
NKT cells CSPG4 CAR vs $CD8^+$ T cells CSPG4 CAR	ns	ns	ns	ns	ns	ns	ns	ns
$CD8^+$ T cells mock vs $CD8^+$ T cells CSPG4 CAR	ns	**	***	***	****	***	****	**

[1] calculated by unpaired student's *t* test from 5–7 independent experiments; **** $p \leq 0.0001$; *** $p \leq 0.001$; ** $p \leq 0.01$; * $p \leq 0.05$; ns $p > 0.1$. *p*-values between 0.05 and 0.1 are specified.

Table S4. *p*-values [1] corresponding to Figure S1.

Conditions	Timepoints							
	**0 h**	**2 h**	**4 h**	**8 h**	**12 h**	**24 h**	**48 h**	**72 h**
NKT cells mock vs NKT cells CSPG4 CAR	ns	*	**	**	**	**	**	**
NKT cells mock vs $CD8^+$ T cells mock	ns	ns	ns	ns	ns	ns	ns	ns
NKT cells CSPG4 CAR vs $CD8^+$ T cells CSPG4 CAR	ns	ns	ns	ns	ns	ns	ns	ns
$CD8^+$ T cells mock vs $CD8^+$ T cells CSPG4 CAR	ns	**	**	**	***	***	****	**

[1] calculated by unpaired student's *t* test from 5–7 independent experiments; **** $p \leq 0.0001$; *** $p \leq 0.001$; ** $p \leq 0.01$; * $p \leq 0.05$; ns $p > 0.1$. *p*-values between 0.05 and 0.1 are specified.

Table S5. *p*-values [1] corresponding to Figure S2B.

Conditions CAR^+	Timepoints							
	**0 h**	**2 h**	**4 h**	**8 h**	**12 h**	**24 h**	**48 h**	**72 h**
NKT cells mock vs NKT cells CSPG4 CAR	ns	****	****	****	****	****	****	****
NKT cells mock vs $CD8^+$ T cells mock	ns	ns	ns	ns	ns	ns	ns	ns
NKT cells CSPG4 CAR vs $CD8^+$ T cells CSPG4 CAR	ns	ns	ns	ns	ns	ns	ns	ns
$CD8^+$ T cells mock vs $CD8^+$ T cells CSPG4 CAR	ns	****	****	****	****	****	****	****

[1] calculated by unpaired student's *t* test from 5–7 independent experiments; **** $p \leq 0.0001$; *** $p \leq 0.001$; ** $p \leq 0.01$; * $p \leq 0.05$; ns $p > 0.1$. *p*-values between 0.05 and 0.1 are specified.

Table S6. *p*-values [1] corresponding to Figure 3.

Conditions	T2.A1			A375M		
	IL-2	TNF	IFNγ	IL-2	TNF	IFNγ
NKT cells CEA CAR vs NKT cells CSPG4 CAR	ns	ns	ns	ns	*	0.0584
NKT cells CEA CAR vs $CD8^+$ T cells CEA CAR	ns	ns	0.0958	*	0.0909	ns
NKT cells CSPG4 CAR vs $CD8^+$ T cells CSPG4 CAR	ns	ns	ns	*	ns	ns
$CD8^+$ T cells CEA CAR vs $CD8^+$ T cells CSPG4 CAR	ns	ns	0.0841	*	*	0.0646

[1] calculated by unpaired student's *t* test from 4–7 independent experiments; * $p \leq 0.05$; ns $p > 0.1$. *p*-values between 0.05 and 0.1 are specified.

Table S7. *p*-values [1] corresponding to Figure 3.

Conditions	IL-2	TNF	IFNγ
T2.A1 NKT cells CEA CAR vs A375M NKT cells CEA CAR	ns	ns	ns
T2.A1 NKT cells CSPG4 CAR vs A375M NKT CSPG4 CAR	0.0815	**	*
T2.A1 $CD8^+$ T cells CEA CAR vs A375M $CD8^+$ T cells CEA CAR	ns	ns	ns
T2.A1 $CD8^+$ T cells CSPG4 CAR vs A375M $CD8^+$ T cells CSPG4 CAR	*	*	0.0547

[1] calculated by unpaired student's *t* test from 4–7 independent experiments; ** $p \leq 0.01$; * $p \leq 0.05$; ns $p > 0.1$. *p*-values between 0.05 and 0.1 are specified.

Table S8. *p*-values [1] corresponding to Figure 4.

Conditions A375M	Effector/Target			
	60:1	20:1	6:1	2:1
NKT cells CEA CAR vs NKT cells CSPG4 CAR	****	***	***	***
NKT cells CEA CAR vs $CD8^+$ T cells CEA CAR	ns	ns	ns	ns
NKT cells CSPG4 CAR vs $CD8^+$ T cells CSPG4 CAR	ns	ns	ns	ns
$CD8^+$ T cells CEA CAR vs $CD8^+$ T cells CSPG4 CAR	0.0997	**	**	*

[1] calculated by unpaired student's *t* test from 4–7 independent experiments; **** $p \leq 0.0001$; *** $p \leq 0.001$; ** $p \leq 0.01$; * $p \leq 0.05$; ns $p > 0.1$. *p*-values between 0.05 and 0.1 are specified.

Table S9. *p*-values [1] corresponding to Figure 4.

Conditions	Effector/Target			
	60:1	20:1	6:1	2:1
T2.A1 NKT cells CEA CAR vs A375M NKT cells CEA CAR	*	ns	ns	ns
T2.A1 NKT cells CSPG4 CAR vs A375M NKT cells CSPG4 CAR	****	****	****	****
T2.A1 $CD8^+$ T cells CEA CAR vs A375M $CD8^+$ T cells CEA CAR	*	**	*	*
T2.A1 $CD8^+$ T cells CSPG4 CAR vs A375M $CD8^+$ T cells CSPG4 CAR	***	****	****	**

[1] calculated by unpaired student's *t* test from 4–7 independent experiments; **** $p \leq 0.0001$; *** $p \leq 0.001$; ** $p \leq 0.01$; * $p \leq 0.05$; ns $p > 0.1$. *p*-values between 0.05 and 0.1 are specified.

Table S10. *p*-values [1] corresponding to Figures 5 and S5.

**Conditions Jurkat**	**Effector/Target**			
	**60:1**	**20:1**	**6:1**	**2:1**
NKT cells mock vs NKT cells CSPG4 CAR	ns	ns	ns	**
NKT cells mock vs CD8+ T cells mock	**	0.0793	ns	ns
NKT cells CSPG4 CAR vs CD8+ T cells CSPG4 CAR	ns	ns	ns	ns
CD8+ T cells mock vs CD8+ T cells CSPG4 CAR	*	ns	ns	ns
Conditions Jurkat + αGalCer	Effector/Target			
	60:1	20:1	6:1	2:1
NKT cells mock vs NKT cells CSPG4 CAR	ns	ns	ns	ns
NKT cells mock vs CD8+ T cells mock	0.0698	*	ns	ns
NKT cells CSPG4 CAR vs CD8+ T cells CSPG4 CAR	ns	ns	ns	ns
CD8+ T cells mock vs CD8+ T cells CSPG4 CAR	ns	0.0687	ns	ns

[1] calculated by unpaired student's *t* test from 4 independent experiments; ** $p \leq 0.01$; * $p \leq 0.05$; ns $p > 0.1$. *p* -values between 0.05 and 0.1 are specified.

Table S11. *p*-values [1] corresponding to Figures 5 and S5.

**Conditions**	**Effector/Target**			
	**60:1**	**20:1**	**6:1**	**2:1**
Jurkat NKT cells mock vs Jurkat+αGalCer NKT cells mock	ns	ns	ns	ns
Jurkat NKT cells CSPG4 CAR vs Jurkat+αGalCer NKT cells CSPG4 CAR	ns	ns	ns	*
Jurkat CD8+ T cells mock vs Jurkat+αGalCer CD8+ T cells mock	*	ns	ns	ns
Jurkat CD8+ T cells CSPG4 CAR vs Jurkat+αGalCer CD8+ T cells CSPG4 CAR	ns	ns	ns	ns

[1] calculated by unpaired student's *t* test from 4 independent experiments; * $p \leq 0.05$; ns $p > 0.1$. *p*-values between 0.05 and 0.1 are specified.

© 2018 by the authors. Submitted for possible open access publication under the terms and conditions of the Creative Commons Attribution (CC BY) license (http://creativecommons.org/licenses/by/4.0/).
